# Impact of metformin use on the recurrence of hepatocellular carcinoma after initial liver resection in diabetic patients

**DOI:** 10.1371/journal.pone.0247231

**Published:** 2021-03-04

**Authors:** Wei-Ru Cho, Chih-Chi Wang, Meng-Yun Tsai, Chen-Kai Chou, Yueh-Wei Liu, Yi-Ju Wu, Ming-Tsung Lin, Kuang-Den Chen, Ching-Hui Chuang, Pao-Yuan Huang, Tsung-Hui Hu, Ming-Chao Tsai

**Affiliations:** 1 Division of Hepato-Gastroenterology, Department of Internal Medicine, Kaohsiung Chang Gung Memorial Hospital and Chang Gung University College of Medicine, Kaohsiung, Taiwan; 2 Department of Surgery, Kaohsiung Chang Gung Memorial Hospital, Chang Gung University Medical College, Kaohsiung, Taiwan; 3 Division of Pulmonary and Critical Care Medicine, Kaohsiung Chang Gung Memorial Hospital, Kaohsiung, Taiwan; 4 Division of Endocrinology and Metabolism, Department of Internal Medicine, Kaohsiung Chang Gung Memorial Hospital and Chang Gung University College of Medicine, Kaohsiung, Taiwan; 5 Center for Translational Research in Biomedical Sciences, Liver Transplantation Program and Department of Surgery, Kaohsiung Chang Gung Memorial Hospital and Chang Gung University College of Medicine, Kaohsiung, Taiwan; 6 Department of Nursing, Meiho University, Pingtung, Taiwan; 7 Graduate Institute of Clinical Medical Sciences, Chang Gung University College of Medicine, Taoyuan, Taiwan; Kaohsiung Medical University Chung Ho Memorial Hospital, TAIWAN

## Abstract

**Background:**

Metformin is proposed to have chemopreventive effect of various cancer currently. However, the anti-cancer effect of metformin for diabetic patients with hepatocellular carcinoma (HCC) undergoing liver resection remains unclear. The aim of our cohort study was to assess whether metformin influence the recurrence of HCC.

**Methods:**

We retrospectively enrolled 857 HCC patients who received primary resection from April 2001 to June 2016. 222 patients were diagnosed with diabetes mellitus (DM) from medical record. Factors influence the overall survival (OS) and recurrence-free survival (RFS) were analyzed by multivariate analysis.

**Results:**

During the follow-up period (mean, 75 months), 471 (54.9%) patients experienced recurrence, and 158 (18.4%) patients died. Multivariate analysis revealed that DM (*p* = 0.015), elevated AST (*p* = 0.006), hypoalbuminemia (*p* = 0.003), tumor number (*p* = 0.001), tumor size (*p* < 0.001), vascular invasion (*p* <0.001), high Ishak fibrosis score (*p* <0.001), hepatitis B (*p* = 0.014), hepatitis C (*p* = 0.001) were independent predictors for RFS. In diabetic patients, only HbA1c>9% (*p* = 0.033), hypoalbuminemia (*p* = 0.030) and vascular invasion (*p* = 0.001) were independent risk factors for HCC recurrence; but the metformin use revealed no significance on recurrence. DM is a risk factor of HCC recurrence after resection. Adequate DM control can reduce the recurrence of HCC. However, the use of metformin does not reduce the risk of HCC recurrence in diabetic patient after initial resection. Hence, metformin may not have protective influences on HCC recurrence in diabetic patients who undergo initial liver resection.

## Introduction

Hepatocellular carcinoma (HCC) is the fifth most common cancer worldwide nowadays, and its incidence is approximately 850,000 new cases per year [1–3]. HCC is often considered to be linked to multiple risk factors, such as infections with hepatitis B virus (HBV) or hepatitis C virus (HCV), alcohol abuse, and metabolic syndrome [4]. Metabolic factor such as obesity and diabetes are associated with increased mortality rates of several cancers [5, 6] and diabetes is also reported as a risk factor for liver, pancreatic, renal, and colon cancers [7, 8]. Therefore, therapeutic intervention for diabetes may lead to prevention of HCC recurrence and may improve survival of diabetic HCC patient after hepatectomy.

Metformin is one of the most frequently prescribed antihyperglycemic drugs and is used as the first-line therapy for type 2 diabetes mellitus (T2DM) in Taiwan. Many previous studies have showed an anticancer effect from metformin in several cancer types with T2DM comorbidity [9, 10]. Patient received curative hepatectomy might ask whether metformin can prevent the recurrence of HCC and have better outcomes. However, the anticancer effect has not been noted in all cancers and remains controversial. Little is known for the anticancer effects of metformin on HCC recurrence and mortality recently.

We therefore evaluated the associations between metformin use and the risk of HCC recurrence and mortality among diabetic patients with HCC after curative resection.

## Patients and methods

### Patients

**Fig 1:**
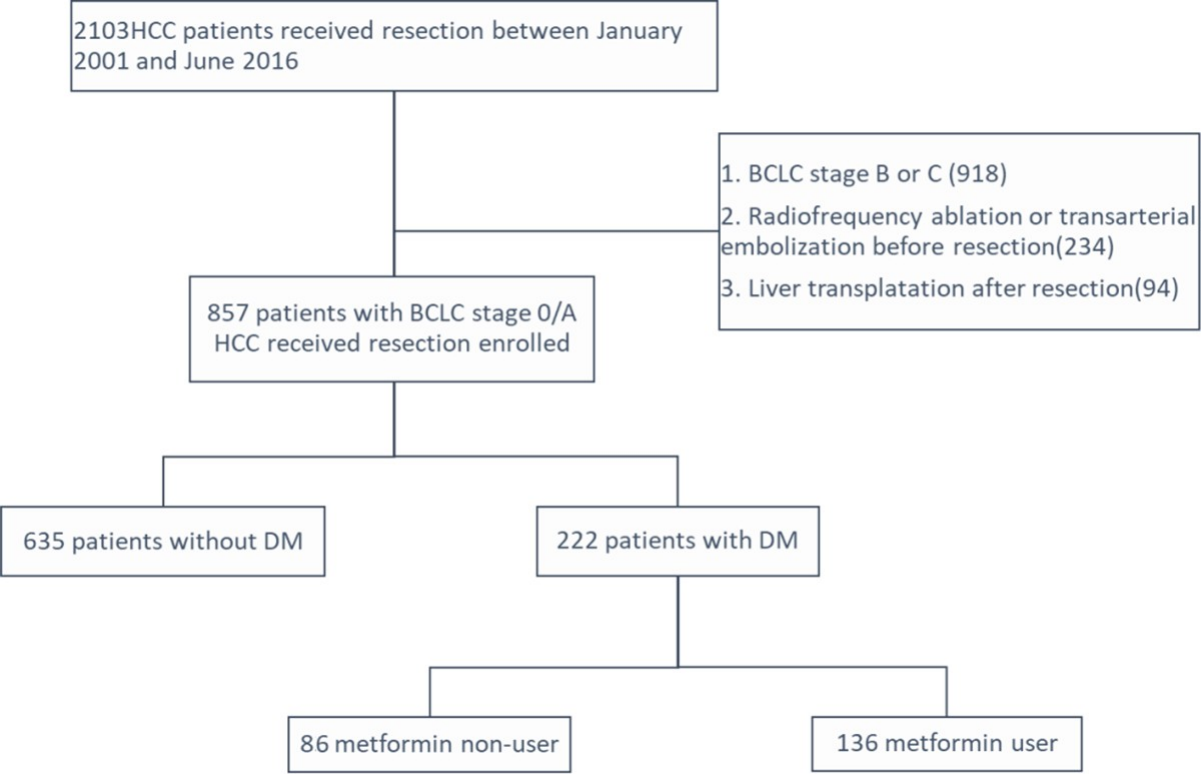
Patient selection flow diagram.

We reviewed a total of 2103 patients who were diagnosed with HCC and underwent surgical resection between January 2001 and June 2016 at Kaohsiung Chang Gung Memorial Hospital. This hospital is a tertiary referral center that covers the southern part of Taiwan. We excluded 234 patients with prior HCC treatment, 918 patients with BCLC stage B or C, 94 patients received liver transplantation after resection. Finally, we recruited 857 patients with BCLC stage 0 or A HCC who underwent primary curative resection. Among them, 222 patients were diagnosed with DM from medical record, and 136 patients used metformin as anti-DM treatment (Fig 1). This study complies with the standards of the Declaration of Helsinki and current ethical guidelines, and approval was obtained from the Ethics Committee of Chang Gung Memorial Hospital. The requirement for informed consent was waived by the IRB (IRB number: 201901103B0).

HCC was defined according to the results of imaging studies and biochemical assays, and the diagnosis was confirmed using histopathology. The HCC diagnosis was based on the criteria of the practice guidelines of the European Association for the Study of the Liver (EASL) or the American Association for the Study of Liver Disease (AASLD) [11, 12]. Patients were included in the T2DM group if they had ≧1 diagnosis of T2DM as noted by an ICD-10 code in the medical record or the usage of anti-diabetic medication for more than 3 months.

### Drug exposure

Drug exposure was defined as receiving OHAs in the same class for at least three months during the follow-up period. All patients treated with metformin were categorized as ‘‘metformin users,” whereas use of other drugs including sulfonylurea, thiazolidinedione, insulin or other OHAs were categorized as ‘‘non-metformin users.” In patients treated with combination therapies, those prescribed metformin for more than 3 months were categorized as metformin users.

### Assessments and follow-up evaluation

The baseline demographics, serum biochemistry, tumor burden and anti-DM therapy were comprehensively recorded before any forms of definite treatment. The diagnosis of cirrhosis, grade of steatosis and Ishak fibrosis score were documented by resected non-tumor pathologic report. The HCC stage was defined according to the Barcelona Clinic Liver Cancer (BCLC) guidelines. Tumor differentiation was determined using the Edmondson grading system. The follow-up ended on November 30, 2019. OS was defined as the interval between the dates of surgery and death or last observation. Patients were followed up at the 1^st^ month after liver resection, followed by every 3 months in the first year and every 3–6 months in subsequent years. Routine tests such as serum AFP levels, serum biochemistry, and abdominal ultrasound were performed at every follow-up. Liver computed tomography or magnetic resonance image were performed at the 1^st^ month after liver section and every 12 months or recurrence was suspected clinically.

### Statistical analysis

Statistical analyses were performed using IDM SPSS Statistics for Windows, version 22.0 (IBM Corp., Armonk, NY, USA). Experimental values of non-continuous variables are expressed as the median ± interquartile range (IQR). The chi-squared test is used as appropriate to evaluate the significance of differences in data and multiple comparison in groups. The relationship between recurrence-free survival (RFS), OS were analyzed using Kaplan–Meier survival curves and the log-rank test, and p<0.050 was considered statistically significant. Factors that were significant in the univariate analysis (p <0.05) were included in a multivariate analysis using a Cox forward stepwise variable selection process of the estimated OS and RFS.

## Results

### Baseline characteristics of the study patients

Table 1 presents the baseline characteristics of the study cohort. The mean follow-up time was 75 months. The sample comprised 670 men and 187 women, and the median age was 60 years at enrollment. As shown in Table 1, 635(74%) patients are non-diabetic and 222(26%) patients are diabetic. Compared to patients without DM, patients with DM were significantly older (p <0.001), lower serum bilirubin at baseline (p <0.001), higher prevalence of hypertension (p <0.001), higher BMI (p <0.001), higher grade of steatosis (p = 0.003), higher prevalence of HCV infection(p = 0.005), lower percentage of HBV infection (p< 0.001) but had a higher percentage of recurrence (p = 0.019). Overall, patients with DM had higher rates of death (28.4%) than subjects without DM (14.9%, p < 0.001).

**Table 1:** Comparison of clinical and pathological characteristics between patients with DM or not before hepatectomy.

	Total (n = 857)	DM (n = 222)	Non DM (n = 635)	P value
Age (years; median, IQR)	60(52~66)	62(57~68)	58.9(50~66)	<0.001
Age (>60 years), n (%)	449 (52.4%)	146 (65.8%)	303 (47.7%)	<0.001
Male, n (%)	670 (78.2%)	170 (76.6%)	500 (78.7%)	0.502
Bilirubin (g/dL; median, IQR)	0.8(0.6~1.0)	0.7(0.5~0.9)	0.8(0.6~1)	<0.001
Albumin (g/dL; median, IQR)	3.7(3.2~4.09)	3.66(3.10~4.09)	3.70(3.30~4.09)	0.177
AST (U/L; median, IQR)	35(26~51)	35(27~52.5)	34(26~50)	0.316
ALT (U/L; median, IQR)	37(26~61)	37(25~64)	37(26~59)	0.714
Creatinine (mg/dL; median, IQR)	0.86(0.73~1.02)	0.87(0.7~1.1)	0.86(0.74~1)	0.228
AFP (>200ng/mL), n (%)	158 (18.4%)	37 (16.7%)	121 (19.1%)	0.462
Liver cirrhosis, n (%)	400 (46.7%)	115 (51.8%)	285 (44.9%)	0.075
Tumor size (>2cm), n (%)	643 (75%)	175 (78.8%)	468 (73.7%)	0.137
Tumor number (single: multiple)	780: 77	199: 23	581: 54	0.405
Child-Pugh grade (A: B)	786: 71	201: 21	585: 50	0.461
Micro/Macrovascular invasion, n (%)	320 (37.3%)	89 (40.1%)	231 (36.4%)	0.325
Histological grade (well: moderate: poor)	110: 714: 22	22: 189: 9	88: 525: 13	0.265
Hypertension, n (%)	321(37.5%)	137(61.7%)	184(28.9%)	<0.001
Smoking, n (%)	242(28.2%)	65(29.3%)	177(27.9%)	0.699
BMI	24.4(22.2~26.8)	25.39(22.83~28)	24.16(22.04~26.28)	<0.001
the grade of steatosis (<5%:5–33%: >33%)	405:307:39	91:107:11	314:200:28	0.003
Ishak fibrosis score (0–3:4–6)	273:419	69:119	204:300	0.366
HBV, n%	484(56.5%)	95(42.8%)	389(61.3%)	<0.001
HCV, n%	300(35%)	95(42.8%)	205(32.3%)	0.005
Recurrence, n (%)	471 (55%)	137 (61.7%)	334 (52.6%)	0.019
Death, n (%)	158 (18.4%)	63 (28.4%)	95 (14.9%)	<0.001

AFP = α-fetoprotein

The chemopreventive effect of medication in diabetic patients with HCC after curative resection

**Fig 2:**
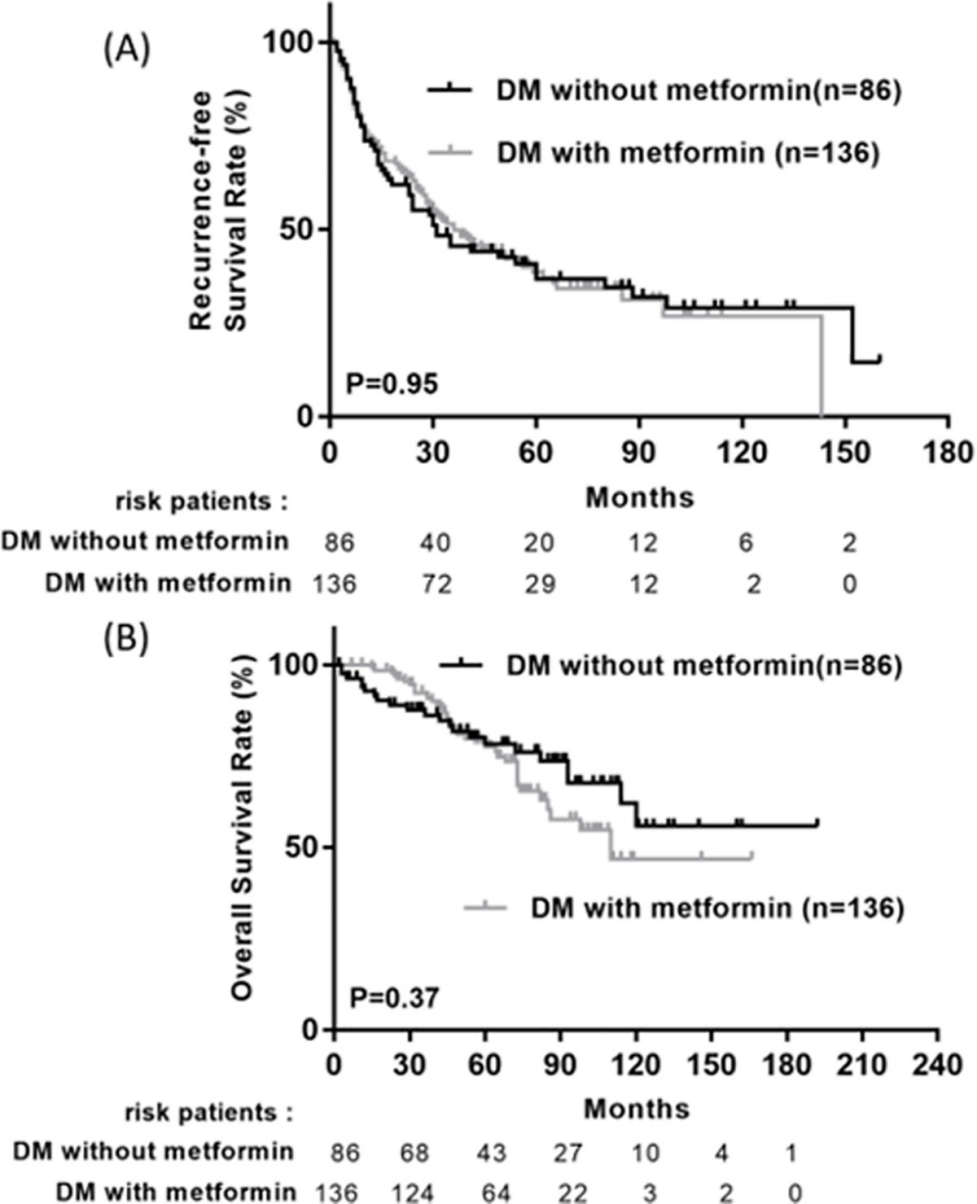
Cumulative recurrence-free survival (A) and overall survival (B) between patients with and without metformin use in diabetic patients.

**Fig 3:**
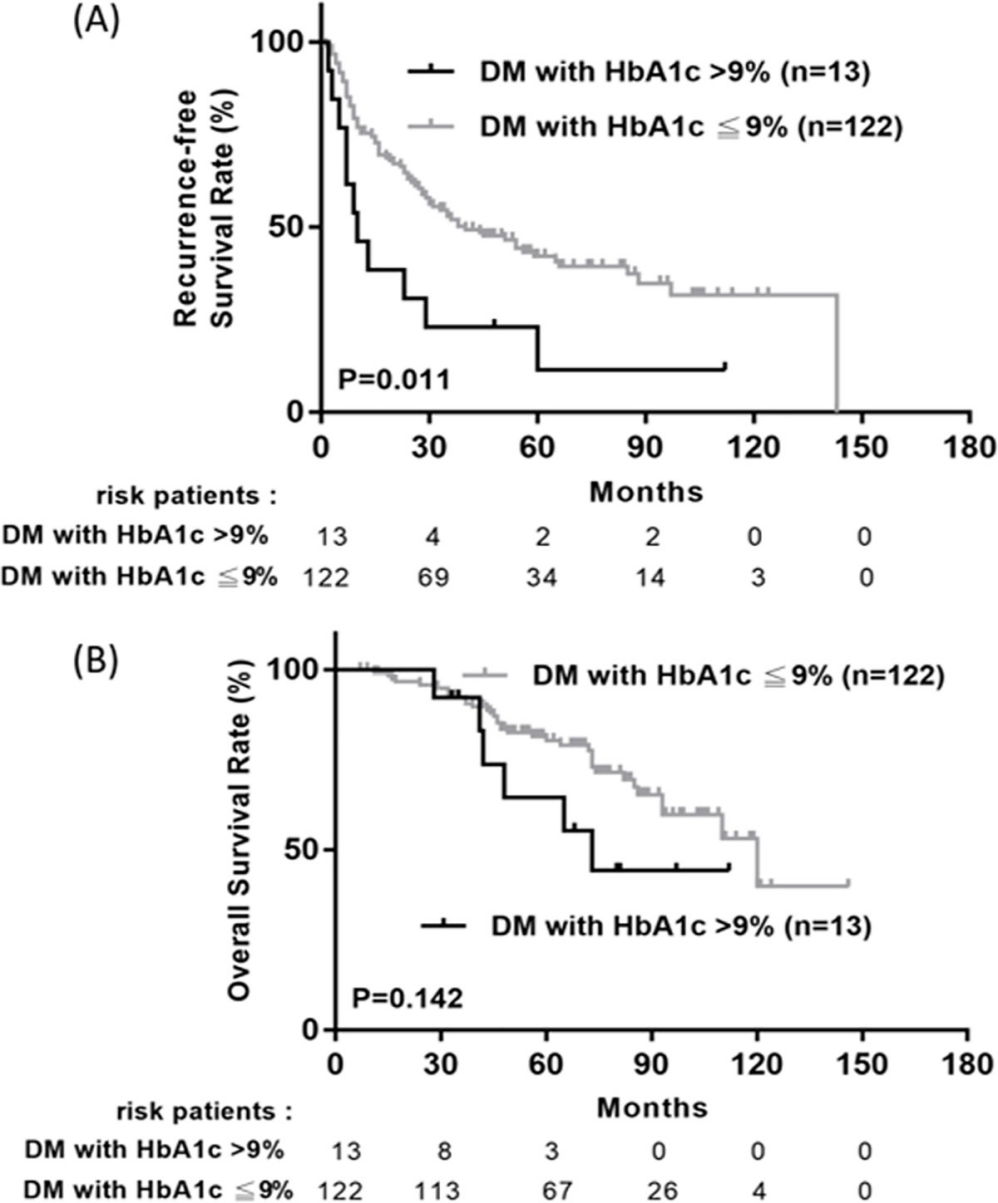
Cumulative recurrence-free survival (A) and overall survival (B) between diabetic with HbA1c>9% and those with HbA1C≦9%.

Among those who with diabetic, 136 patients are metformin users and eighty-six are non-metformin users. Kaplan-Meier analysis reveals no statistically significant in overall survival and recurrence free survival between metformin group and non-metformin group (Fig 2A and 2B). Poor DM control (defined as HbA1C> 9%) would lead to higher recurrence rate of HCCs (p = 0.011) and a trend of poor overall survival (p = 0.142) (Fig 3A and 3B).

### Independent factors for HCC recurrence

**Fig 4:**
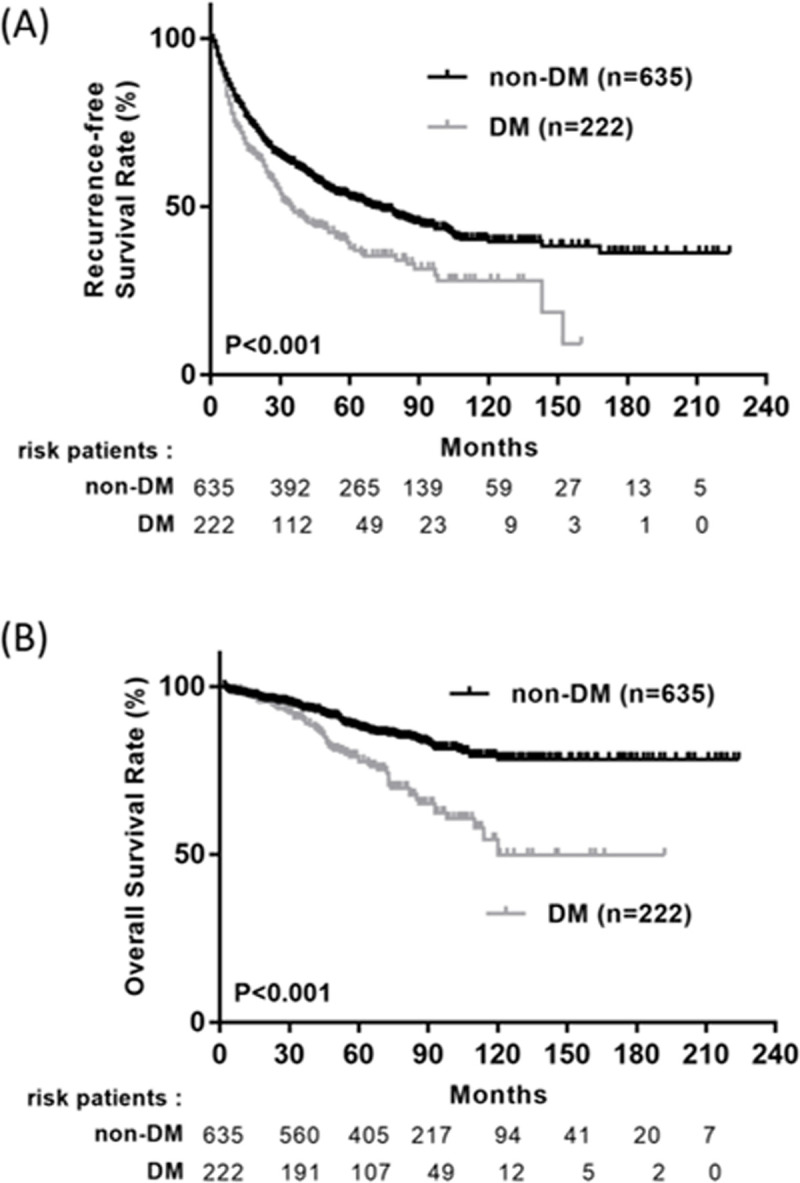
Long-term outcomes between patients with and without diabetes mellitus. (A) Recurrence-free survival; (B) Overall survival.

As shown in Table 2, age, diabetes mellitus (DM), hypertension, AST, ALT, thrombocytopenia, hypoalbuminemia, liver cirrhosis, Child Pugh grade, tumor number, tumor size, histology stage, vascular invasion, higher Ishak fibrosis score, hepatitis C were significantly associated with HCC recurrence in univariate analysis. In the multivariate analysis, DM (hazard ratio [12], 1.357; 95% CI, 1.061–1.735; *p* = 0.015), AST>40 (hazard ratio [12], 1.356; 95% CI, 1.090–1.687; *p* = 0.006), albumin≦3 (HR, 1.460; 95% CI, 1.140–1.869; *p* = 0.003), tumor number >1 (HR, 1.731; 95% CI, 1.236–2.425; *p* = 0.001), tumor size (HR, 1.298; 95% CI, 1.170–1.441; *p* < 0.001), and vascular invasion (HR, 1.631; 95% CI, 1.302–2.041; *p* < 0.001), Ishak fibrosis score >3 (HR, 1.730; 95% CI, 1.356–2.208; *p* < 0.001), hepatitis B (HR, 1.561; 95% CI, 1.093–2.229; *p* = 0.014), hepatitis C (HR, 1.866; 95% CI, 1.308–2.662; *p* = 0.001) remained independent predictive factors for RFS. The overall survival and RFS were significantly higher among patient without diabetic compared with T2DM patients during the follow-up period (Fig 4A and 4B).

**Table 2:** Univariate and multivariate analyses for recurrence in BCLC 0/A patients with HCC after curative hepatectomy.

			Univariate		Multivariate	
Variable	Comparison	N (%)	HR (95%CI)	P value	HR (95%CI)	P value
Age (years)	> 60	449 (52.4)	1.333 (1.110–1.599)	0.002		
	≦ 60	408 (47.6)				
Sex	Male	670 (78.2)	1.012 (0.812–1.263)	0.913		
	Female	187 (21.8)				
DM	Yes	222 (25.9)	1.449 (1.186–1.770)	<0.001	1.357 (1.061–1.735)	0.015
	No	635 (74.1)				
Hypertension	Yes	321 (37.5)	1.350 (1.072–1.171)	0.011		
	No	536 (62.5)				
Smoking	Yes	242 (28.2)	1.149 (0.864–1.528)	0.340		
	No	615 (71.8)				
Alcohol	Yes	182 (21.2)	0.948 (0.691–1.300)	0.741		
	No	675 (78.8)				
BMI	>25	360 (43.5)	1.033 (0.821–1.299)	0.784		
	≦25	468 (56.5)				
AST(U/L)	>40	320 (37.4)	1.727 (1.439–2.072)	<0.001	1.356 (1.090–1.687)	0.006
	≦40	536 (62.6)				
ALT (U/L)	>40	372 (43.7)	1.469 (1.225–1.762)	<0.001		
	≦40	479 (56.3)				
Total bilirubin	per mg/dL		0.986 (0.604–1.610)	0.956		
Serum creatinine	per mg/dL		0.978 (0.912–1.049)	0.533		
AFP (ng/mL)	> 200	158 (19)	1.053 (0.829–1.337)	0.673		
	≦ 200	674 (81)				
Platelet (10^9^/L)	≦ 150	439 (52)	1.515 (1.261–1.819)	<0.001		
	>150	406 (48)				
Albumin (g/dL)	≦3	160 (18.8)	1.770 (1.425–2.199)	<0.001	1.460 (1.140–1.869)	0.003
	>3	689 (81.2)				
Liver cirrhosis	Yes	400 (46.7)	1.673 (1.395–2.007)	<0.001		
	No	457 (53.3)				
Child-Pugh grade	B	71 (8.3)	1.409 (1.002–1.943)	0.036		
	A	786 (91.7)				
Tumor no.	Multiple	77 (9)	1.727 (1.306–2.284)	<0.001	1.731 (1.236–2.425)	0.001
	Single	780 991)				
Tumor size (cm)	per centimeter		1.245 (1.155–1.800)	0.001	1.298 (1.170–1.441)	<0.001
Histology stages	poor	22 (2.6)	2.561 (1.598–4.106)	<0.001		
	well +moderate	824 (97.4)				
Vascular invasion*	Yes	320 (37.3)	1.582 (1.314–1.903)	<0.001	1.631 (1.302–2.041)	<0.001
	No	537 (62.7)				
Grade of steatosis	<5% (Reference)	405 (53.9)				
	5–33%	307 (40.9)	1.129 (0.895–1.425)	0.307		
	>33%	39 (5.2)	0.895 (0.502–1.596)	0.707		
Ishak fibrosis score	4–6	419 (60.5)	1.787 (1.416–2.256)	<0.001	1.730 (1.356–2.208)	<0.001
	0–3	273 (39.5)				
Hepatitis B	Yes	484 (56.5)	1.148 (0.957–1.377)	0.136	1.561 (1.093–2.229)	0.014
	No	373 (43.5)				
Hepatitis C	Yes	300 (35)	1.422 (1.182–1.711)	<0.001	1.866 (1.308–2.662)	0.001
	No	557 (65)				
Surgery	minor	602 (70.2)	0.889 (1.689–1.147)	0.364		
	major	255 (29.8)				
	Conventional	757 (88.3)	0.728 (0.518–1.023)	0.067		
	laparoscopic	100 (11.7)				

*2 patients are macrovascular invasion.

In subgroup of diabetic patients, higher HbA1c, AST>40(U/L), ALT>40(U/L), thrombocytopenia, hypoalbuminemia, liver cirrhosis and vascular invasion were significantly associated with RFS in univariate analysis. In the multivariate analysis, HbA1C >9% (HR, 2.095; 95% CI, 1.061–4.139; *p* = 0.033), hypoalbuminemia (HR, 1.883; 95% CI, 1.065–3.330; *p* = 0.030), vascular invasion (HR, 2.586; 95% CI, 1.473–4.537; *p* = 0.001) remained independent predictive factors for RFS (Table 3).

**Table 3:** Univariate and multivariate analysis for recurrence in BCLC 0/A patients with DM after curative hepatectomy.

			Univariate		Multivariate	
Variable	Comparison	N (%)	HR (95%CI)	P value	HR (95%CI)	P value
Age (years)	> 60	146 (65.8)	1.173 (0.830–1.658)	0.365		
	≦ 60	76 (34.2)				
Sex	Male	170 (76.6)	1.011 (0.676–1.513)	0.957		
	Female	52 (23.4)				
HbA1C(%)	>9	13 (9.6)	2.220 (1.174–4.196)	<0.014	2.095 (1.061–4.139)	0.033
	≦ 9	122 (90.4)				
Metformin	Yes	136 (61.3)	0.991 (0.696–1.410)	0.958		
	No	86 (38.7)				
Sulfonylurea	Yes	103 (46.4)	0.994 (0.708–1.395)	0.971		
	No	119 (53.6)				
Insulin	Yes	28 (12.6)	1.591 (0.984–2.572)	0.058		
	No	194 (87.4)				
Hypertension	Yes	137 (60.9)	1.186 (0.775–1.813)	0.432		
	No	85 (39.1)				
Smoking	Yes	65 (29.3)	1.384 (0.847–2.261)	0.195		
	No	157 (70.7)				
Alcohol	Yes	54 (24.3)	0.735 (0.423–1.278)	0.276		
	No	168 (75.7)				
BMI (kg/m^2^)	>25	119 (54.3)	0.829 (0.531–1.296)	0.411		
	≦25	100 (45.7)				
AST(U/L)	>40	93 (40.1)	1.899 (1.354–2.663)	<0.001		
	≦40	128 (59.9)				
ALT(U/L)	>40	101 (46.3)	1.660 (1.181–2.335)	0.004		
	≦40	117 (53.7)				
Total bilirubin	per mg/dL		0.657 (0.348–1.238)	0.194		
Serum creatinine	per mg/dL		0.990 (0.805–1.219)	0.928		
AFP (ng/mL)	> 200	37 (17.3)	1.415 (0.908–2.204)	0.125		
	≦ 200	177 (82.7)				
Platelet (10^9^/L)	≦ 150	110 (50)	1.585 (1.126–2.232)	0.008		
	> 150	110 (50)				
Albumin (g/dL)	≦3	47 (21.6)	1.717(1.155–2.554)	0.008	1.883 (1.065–3.330)	0.030
	>3	171 (88.4)				
Liver cirrhosis	Yes	115 (51.8)	1.457 (1.038–2.045)	0.029		
	No	107 (48.2)				
Child-Pugh grade	B	21 (9.5)	1.326 (0.732–2.403)	0.352		
	A	201 (90.5)				
Tumor no.	Multiple	23 (10.4)	1.383 (0.831–2.301)	0.212		
	Single	199 (89.6)				
Tumor size (cm)	per centimeter		1.214 (0.796–1.851)	0.368		
Histology stages	Poor	9 (4.1)	1.630 (0.760–3.493)	0.209		
	well + moderate	211 (95.9)				
Vascular invasion	Yes	89 (40.1)	1.588 (1.130–2.232)	0.008	2.586 (1.473–4.537)	0.001
	No	133 (59.9)				
Hepatitis B	Yes	95 (42.8)	1.371 (0.979–1.920)	0.066		
	No	127 (57.2)				
Hepatitis C	Yes	95 (42.8)	1.061 (0.753–1.496)	0.753		
	No	127 (57.2)				
Steatosis grade	<5% (Reference)	91 (43.5)				
	5–33%	107 (52.2)	1.659 (1.042–2.643)	0.033		
	>33%	11 (4.3)	0.459 (0.107–1.971)	0.295		
Ishak fibrosis score	4–6	119 (63.3)	1.297 (0.841–1.998)	0.239		
	0–3	69 (36.7)				
Surgery	major	59 (26.6)	0.846 (0.508–1.411)	0.522		
	minor	163 (73.4)				
	Conventional	186 (83.8)	0.732 (0.421–1.271)	0.267		
	laparoscopic	36 (16.2)				

### Patterns of recurrence

There are 471 patients with recurrence, 137 DM patients and 334 non-DM patients. The patterns of recurrence among DM patients vs. Non-DM patients are 115(83.9%) versus 273(81.7%) within the Milan criteria (*p* = 0.568); 133(97%) vs. 323(96.7%) with intrahepatic recurrence (*p* = 0.834) and 83(60.6%) vs. 193(57.8%) with early recurrence (*p* = 0.575). The recurrence patterns showed no statistically difference between DM and non-DM patients.

Among 137 DM patients with recurrence, we further divided them into the Metformin users (85 patients) vs. Non-Metformin users (52 patients). The recurrence patterns of these two groups are 70(82.4%) vs. 45(86.5%) within the Milan criteria (*p* = 0.517); 82(96.5%) vs. 51(98%) with intrahepatic recurrence (*p* = 0.588) and 48(56.5%) vs. 35(67.3%) with early recurrence (*p* = 0.208).

### Independent factors for mortality

A total of 63 patients died during the follow-up period; 29 of them suffered from liver-related death: 26 died of HCC and 3 of complications associated with cirrhosis. Of the 34 patients with non-liver-related death, 25 died of sepsis, three of cardiovascular disease, two of malignancies other than HCC and four of DM complications. In the OS analysis, the multivariate Cox proportional hazards model revealed that insulin use (HR, 2.615; 95% CI, 1.424–4.804; *p* = 0.002), albumin≦3.5g/dL (HR, 2.215; 95% CI, 1.321–3.714; *p* = 0.003), vascular invasion (HR, 1.754; 95% CI, 1.066–2.886; *p* = 0.027) were independent risk factors associated with overall mortality. As regards to liver-related death, baseline AFP>200 (HR, 5.050; 95% CI, 1.694–15.056; *p* = 0.004) is poor independent factor.

### Impact of DM and metformin on the outcomes of patients between patients with BCLC stage 0 and A

There are 124 patients categorized to BCLC stage 0 and 733 patients BCLC stage A. Kaplan-Meier plot of overall survival between BCLC 0 and A reveal p = 0.103 without statistically significance. However, the Kaplan-Meier plot of RFS between BCLC 0 and A showed there is statistically significance between these two groups with p<0.001 (S1 Fig). The BCLC 0 group had better RFS than BCLC A.

We further divided our study cohort into BCLC 0 and BCLC A. Kaplan-Meier plots revealed patients with DM had poor overall survival than those without DM in BCLC 0 group(p = 0.012), poor outcomes in RFS and OS in BCLC A (p< 0.001 and <0.001). Metformin wound not affect the outcomes in BCLC 0 and BCLC A (S2 and S3 Figs).

## Discussion

Diabetes is associated with increased mortality rates of several cancers [5, 6] and reported as a risk factor for hepatocellular carcinoma [13]. Also, diabetic patients had higher recurrence rate and poor prognosis compared with those without DM after HCC treatment [14]. Therefore, whether DM management would be beneficial to the prognosis of HCC patients after curative hepatectomy is an important issue and needs further evaluation and studies.

Our study demonstrated that patients with diabetes mellitus have higher recurrence rate and poor overall survival rate after HCC resection compared with those without DM. The finding is identical to the result of Ikeda, Y. *et al* [14]. Diabetic patients have much more comorbidities, increased infection risk, difficult cell regeneration and wound healing, higher risk of cardiovascular events, weakened immune system and lead to poor overall survival. Also, hyperglycemia induces DNA damage and cytotoxicity, which contributed to carcinogenesis [15]. Furthermore, patients with noninsulin dependent diabetes mellitus are characterized by insulin resistance, compensatory hyperinsulinemia and increased growth factor production, which will interact with liver cells and stimulates mitogenesis or carcinogenesis [16, 17].

It is worth noting that in the present study, the use of Metformin is not significant in RFS and OS in diabetic HCC patients after curative resection (p>0.05) (Fig 3A and 3B). There is no statistically significant difference in the clinical and pathological characteristics between metformin and non-metformin user before received curative resection in our study cohort (S1 Table), including the level of glycohemoglobin (p = 0.627). Although many studies and systemic reviews showed the chemopreventive effect of metformin in several cancers as well as in HCC [18–22], some studies also demonstrated metformin doesn’t improve the survival in patients with hepatocellular carcinoma [23] and doesn’t reduce the risk of HCC in diabetic patients [24]. The reasons might be explained that although diabetes mellitus is a progressive disease accompanied by persistent chronic inflammation results from hyperglycemia or hyperinsulinemia, which play key roles in cancer cell activity, including its initiation, promotion, and progression [25], metformin can decrease insulin resistance but cannot directly reduce abnormal insulin secretion. In addition, hyperinsulinemia wound directly affect liver tissue and lead to the genesis of HCC but metformin wound not directly inhibit this pathway. Furthermore, DM results from chronic inflammation and can cause additional oxidative stress and lead to the HCC, but the anti-oxidative stress effect of metformin may be too weak to reverse this condition.

To show the dose-dependent relationship, we stratified the study population by metformin daily use level into three groups (non-users, 500–1000 mg, and >1000mg daily dose). The Kaplan-Meier survival analysis showed no statistically significances among non-users and different daily dosage of metformin use in RFS (p = 0.958) and OS (p = 0.355), respectively (S4 Fig). We further stratified the patients by overall metformin use levels into three groups (<90, 90–365, and >365 cDDD). Similarly, there were no significant differences in patients with different cDDD of metformin use in RFS (p = 0.284) as well as the OS (p = 0.606) (S5 Fig). This result implies that there was no dose-dependent relationship between the metformin use and HCC recurrence. However, such analysis was limited by the low number and heterogeneity of the study population. Thus, large, randomized trials in well-selected patients treated with different dosage are warranted to confirm the value of metformin in HCC recurrence.

We further divided our study cohort into BCLC 0 and BCLC A. DM was a poor factor for OS in BCLC 0 group(p = 0.012), and patients without DM had better RFS and OS in BCLC A (p< 0.001 and <0.001). Metformin wound not affect the outcomes in BCLC 0 and BCLC A. Patients in BCLC 0 group had better RFS (p< 0.001) than BCLC A. Therefore, a noninvasive diagnostic strategy to detect HCC at an early stage and to monitor HCC recurrence such as circulating tumor DNA (ctDNA) may provide better outcomes in early HCC patients.

The use of insulin is a risk factor for poor OS and RFS. We noticed the study group of insulin user had higher mortality rate in diabetic patient after HCC resection (p = 0.001). The insulin group had higher glycohemoglobin level (8.25% vs 6.7%, p = 0.013), higher mortality rate (50% vs 25.3%, p = 0.007) and lower albumin level (3.3 vs 3.7, p = 0.012), showed in S2 Table. There is no statistically significant difference in age, gender, liver cirrhosis, Child Pugh grade, tumor size, tumor recurrence between these two study groups. Hyperglycemia contributed to the environment of hyperinsulinemia and increased the demand of insulin for sugar control, which led to a vicious cycle.

Adequate blood sugar control is a good factor for diabetic HCC patients with BCLC 0/A received curative resection. In our present study, patients with poor DM control (HbA1c> 9%) have higher HCC recurrence rate (*p* = 0.011). On the contrary, patients with diabetes under adequate blood sugar control had no difference in HCC recurrence and mortality compared with those without DM. These results indicated that adequate management of hyperglycemia led to reduction in the risk of HCC recurrence and improvement of overall survival. Hyperglycemia and hyperinsulinemia cause a chronic inflammation condition and lead to the genesis of cancer cell. If we can well control the blood sugar of diabetic patient, which would not lead to vicious course of hyperinsulinemia, cause chronic inflammation and oxidative stress. Hosokawa et al emphasized that inadequate maintenance of blood glucose in diabetic patients is a significant risk factor for recurrence of HCC and for poor survival after curative RFA therapy [26]. Therefore, we suggested diabetic patient should focus on adequate blood sugar maintenance rather than craving for the chemopreventive effect of metformin in HCC. There may be several mechanisms involved in the relationship between hyperglycemia and HCC recurrence. In animal study [27], high sugar content diet leads to the greatest liver tumor incidence. Diet-induced postpradial hyperglycemia and hyperinsulinemia significantly correlated with tumor incidence. Hyperglycemia promotes cancer cell proliferation [28–30] through accelerated cell cycle progression or through the production of reactive oxygen species. Iwasaki et al. confirmed that high glucose alone, as well as in combination with pro-inflammatory cytokines, could stimulate the nuclear factor Kappa-B-mediated transcription in hepatocytes in vitro [31]. The results support our finding, sugar control is the key point to avoid HCC recurrence and overall survival instead of the chemopreventive effect of metformin in diabetic patients. Second, the insulin user’s HbA1c level is higher than non-insulin user, and difficult sugar control, more diabetic complications and shorter survival rate. Also, the use of insulin contributes to hyperinsulinemia and attributes to carcinogenesis. It is compatible with our result, insulin users had poor prognosis after curative hepatectomy.

There are 484 patients with hepatitis B virus infection and 264 patients received nucleos(t)ide analogue (NUC). Also, there are 300 patients with HCV infection and 123 patients received HCV treatment. Kaplan-Meier plots revealed the treatment of HBV and HCV wound lead to better outcomes in OS and RFS.

We further stratified our study cohort based on the viral infection status. Multivariate analysis also revealed the treatment of HBV (HR, 0.601; 95% CI, 0.441–0.818; p = 0.001) and HCV (HR, 0.467; 95% CI, 0.328–0.664; p<0.001) are good independent factor for RFS in each group, the same results as previous study [32–34].

In our study cohort, posthepatectomy liver failure (PHLF) was defined according to by the International Study Group of Liver Surgery (ISGLS) definition [35]. The rate of posthepatectomy liver failure among our study cohort was 6.2% (53/857). 41 patients (41/635, 6.5%) without DM had post hepatectomy liver failure, and 5.4% with the diagnosis of DM had post hepatectomy liver failure. The p value between DM and non-DM is 0.576. As for the subgroup of metformin user and non-metformin user, the metformin user group had higher rates of post hepatectomy liver failure, 8.1% (11/136) vs. 1.2% (1/86) with *p* value = 0.026.

There are some possible limitations in our study. First, it is not a prospective study. However, we believed that the bias was small because patients were followed by the same physicians throughout the course of disease, with clinical and laboratory assessment and HCC screening using ultrasonography every 3–6 months. Second, the prevalence of DM in Taiwan is 6.6%, whereas it is up to 12.3% or more in the population of the Western countries [36]. Moreover, the access to medical professionals of blood sugar control is easy and affordable in Taiwan but medication nonadherence remains must not to be ignored.

In conclusion, DM is a risk factor of HCC recurrence after resection. Adequate blood sugar control is associated with the prognosis of diabetic patients with BCLC 0/A HCC after curative resection. However, the use of metformin does not reduce the risk of HCC recurrence in diabetic cohort after initial resection. Hence, we suggested diabetic patient with HCC after resection should go on adequate diet and/or medication control for blood sugar maintenance rather than craving for the chemopreventive effect of metformin in HCC. Further prospective randomized controlled study is required to validate our observation.

## Supporting information

S1 Fig The OS and RFS of BCLC 0 and BCLC A. (TIF)

S2 Fig The OS and RFS of DM and non-DM; metformin and non-metformin users among BCLC stage 0. (TIF)

S3 Fig The OS and RFS of DM and non-DM; metformin and non-metformin users among BCLC stage A. (TIF)

S4 Fig The RFS and OS of DM patient with different daily metformin dosage. (TIF)

S5 Fig The RFS and OS of DM patient with different cDDD of metformin. (TIF)

S1 Table Comparison of clinical and pathological characteristics between DM patients with metformin or non-metformin user before hepatectomy. (DOCX)

S2 Table Comparison of clinical and pathological characteristics between DM patients with insulin or non-insulin user before hepatectomy. (DOCX)

## Abbreviations

DM: diabetes mellitus
HCC: hepatocellular carcinoma
HbA1c: hemoglobin A1c
