# Supplementary figures and images for: Impact of metformin use on the recurrence of hepatocellular carcinoma after initial liver resection in diabetic patients
Source: PLoS One. 2021 Mar 4;16(3):e0247231. doi: 10.1371/journal.pone.0247231 (PMC7932176; doi:10.1371/journal.pone.0247231)

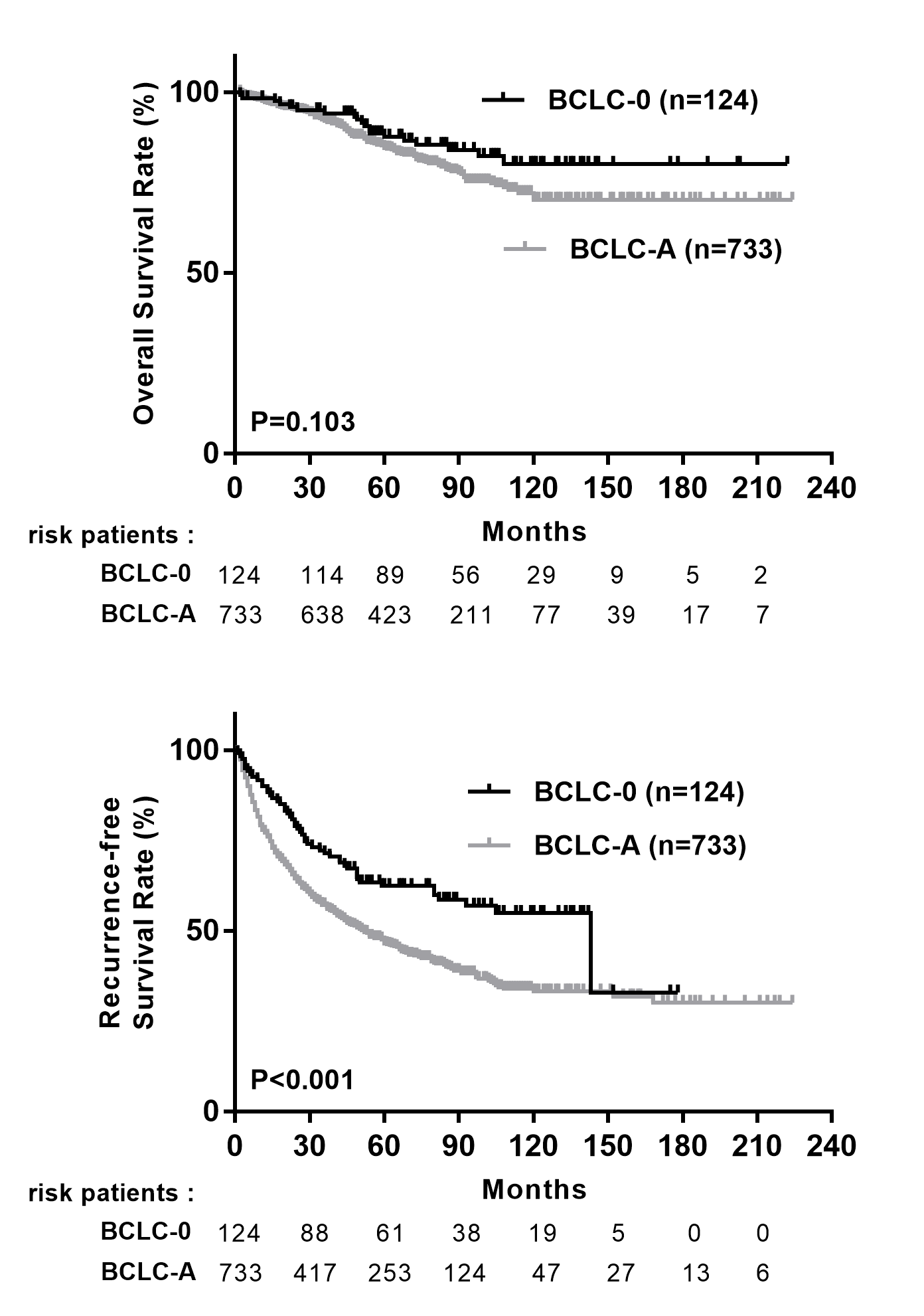

Supplement: S1 Fig — (TIF) [file pone.0247231.s001.tif]

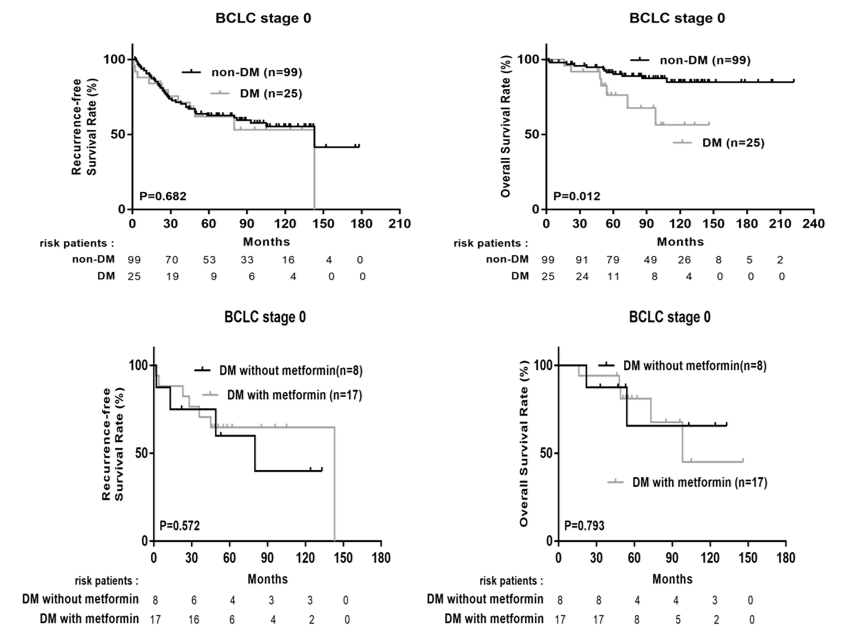

Supplement: S2 Fig — (TIF) [file pone.0247231.s002.tif]

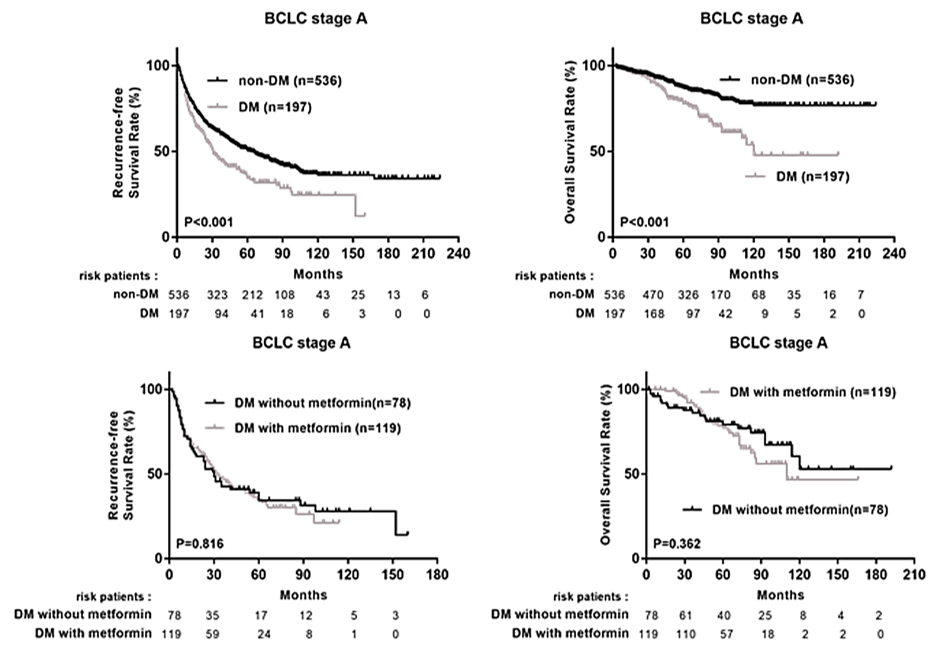

Supplement: S3 Fig — (TIF) [file pone.0247231.s003.tif]

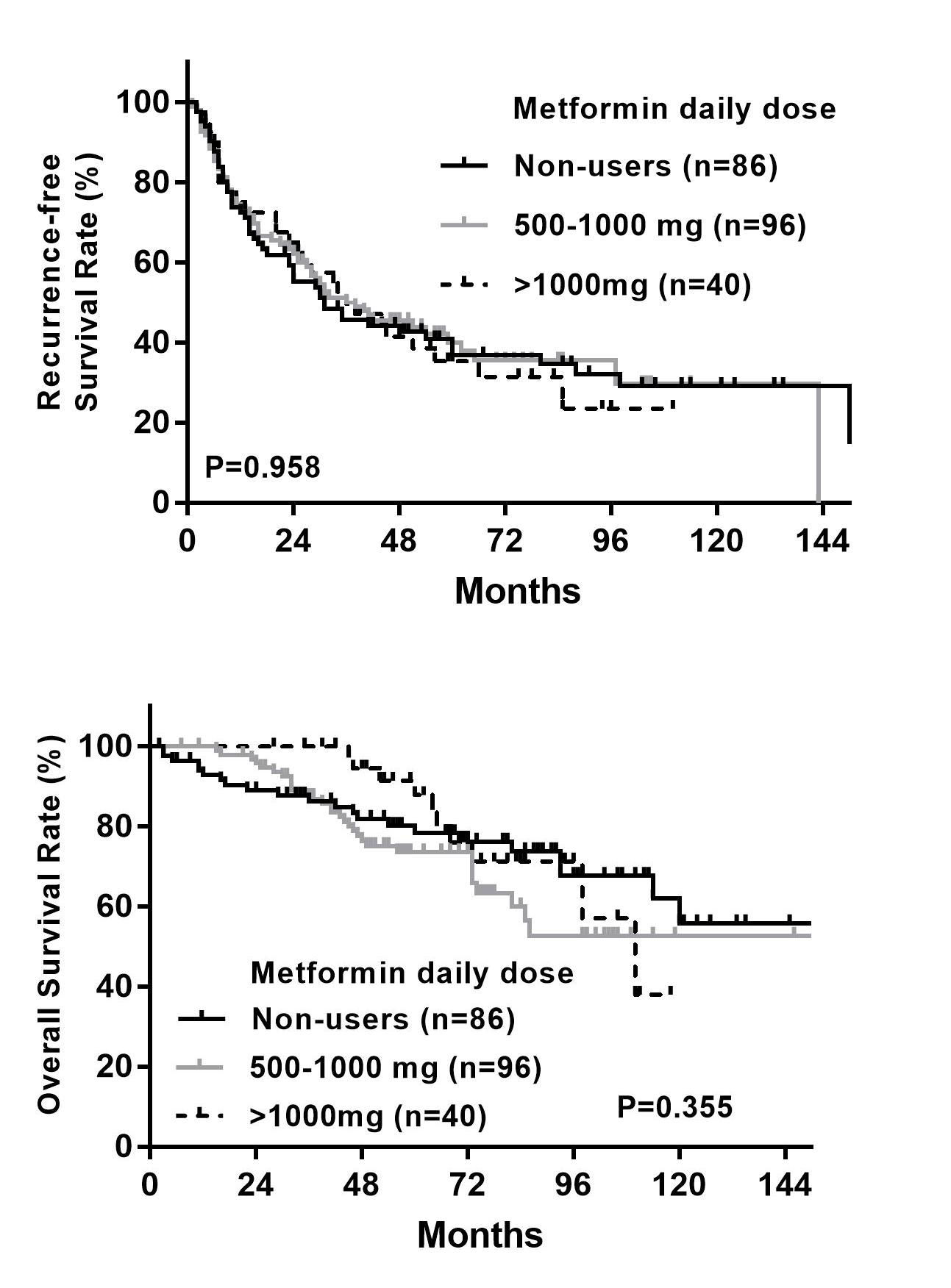

Supplement: S4 Fig — (TIF) [file pone.0247231.s004.tif]

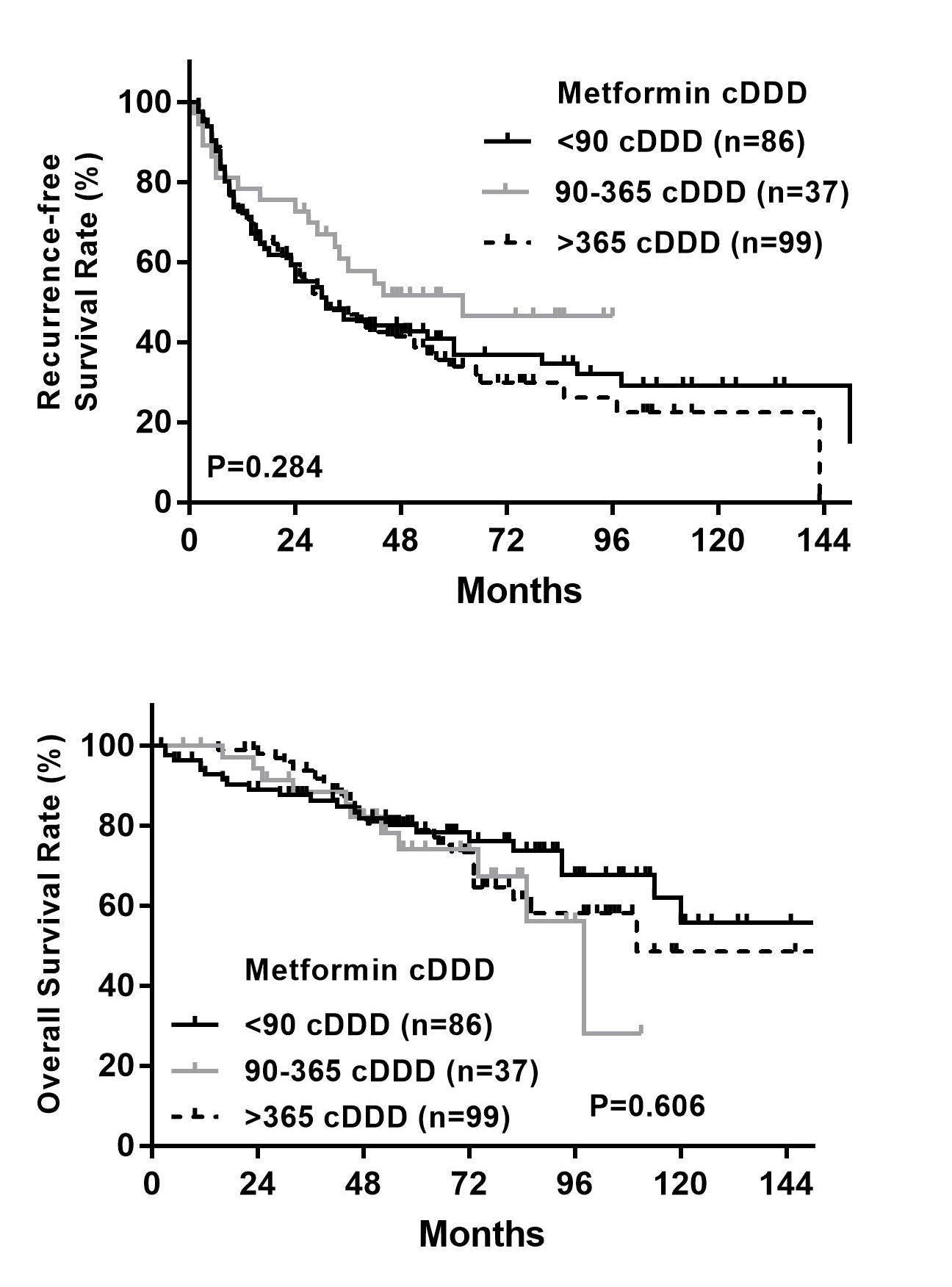

Supplement: S5 Fig — (TIF) [file pone.0247231.s005.tif]
