# Supplementary material for: Impact of metformin use on the recurrence of hepatocellular carcinoma after initial liver resection in diabetic patients
Source: PLoS One. 2021 Mar 4;16(3):e0247231. doi: 10.1371/journal.pone.0247231 (PMC7932176; doi:10.1371/journal.pone.0247231)
Supplement: S1 Table — (DOCX) [file pone.0247231.s006.docx]

**S1 Table. Comparison of clinical and pathological characteristics between DM patients with metformin or non-metformin user before hepatectomy**

|  | Total  (n = 222) | Metformin user  (n = 136) | Non Met user  (n = 86) | P value |
| --- | --- | --- | --- | --- |
| Age (years; median, IQR) | 62(57~68) | 62(58~68) | 61.78(54.9~61.8) | 0.380 |
| Age (>60 years), n (%) | 146 (65.8%) | 93 (63.7%) | 53 (36.6%) | 0.301 |
| Male, n (%) | 170 (76.6%) | 106 (62.4%) | 64 (37.6%) | 0.546 |
| Bilirubin (g/dL; median, IQR) | 0.7(0.5~0.9) | 0.7(0.6~0.9) | 0.7(0.5~1) | 0.706 |
| Albumin (g/dL; median, IQR) | 3.65(3.10~4.10) | 3.70(3.10~4.10) | 3.55(3.00~4.03) | 0.216 |
| HbA1C | 6.8(6.30~7.70) | 6.70(6.28~7.55) | 6.8(6.3~7.85) | 0.627 |
| AFP ( >200ng/mL), n (%) | 37 (16.7%) | 21 (56.8%) | 16 (43.2%) | 0.630 |
| Liver cirrhosis, n (%) | 115 (51.8%) | 69 (60.0%) | 46 (40.0%) | 0.689 |
| Tumor size (>2cm), n (%) | 175 (78.8%) | 106 (60.6%) | 69 (39.4%) | 0.684 |
| Tumor number (single : multiple) | 199 : 23 | 121 : 15 | 78 : 8 | 0.681 |
| Child-Pugh grade (A : B) | 201 : 21 | 121 : 15 | 80 : 6 | 0.315 |
| Micro/Macrovascular invasion, n (%) | 89 (40.1%) | 58 (65.2%) | 31 (34.8%) | 0.328 |
| Histological grade (well : moderate : poor) | 22 : 189 : 9 | 10 : 117 : 7 | 12 : 72 : 2 | 0.186 |
| Recurrence, n (%) | 137 (61.7%) | 85 (62.0%) | 52 (38.0%) | 0.761 |
| Death, n (%) | 63 (28.4%) | 40 (63.5%) | 23 (36.5%) | 0.668 |

AFP = α-fetoprotein
