# Supplementary material for: Impact of metformin use on the recurrence of hepatocellular carcinoma after initial liver resection in diabetic patients
Source: PLoS One. 2021 Mar 4;16(3):e0247231. doi: 10.1371/journal.pone.0247231 (PMC7932176; doi:10.1371/journal.pone.0247231)
Supplement: S2 Table — (DOCX) [file pone.0247231.s007.docx]

**S2 Table. Comparison of clinical and pathological characteristics between DM patients with insulin or non-insulin user before hepatectomy**

|  | Total  (n = 222) | Insulin user  (n = 28) | Non insulin user  (n = 194) | P value |
| --- | --- | --- | --- | --- |
| Age (years; median, IQR) | 62(57~68) | 61.6(54~69.8) | 62(57~68) | 0.515 |
| Age (>60 years), n (%) | 146 (65.8%) | 16 (57.1%) | 130 (67.0%) | 0.304 |
| Male, n (%) | 170 (76.6%) | 21 (75.0%) | 149 (76.8%) | 0.833 |
| Bilirubin (g/dL; median, IQR) | 0.7(0.5~0.9) | 0.7(0.5~1.08) | 0.7(0.5~0.9) | 0.824 |
| Albumin (g/dL; median, IQR) | 3.66(3.10~4.10) | 3.3(2.83~3.80) | 3.7(3.1~4.10) | 0.012 |
| HbA1C | 6.80(6.30~7.70) | 8.25(6.55~10.13) | 6.70(6.30~7.50) | 0.013 |
| AFP ( >200ng/mL), n (%) | 37 (17.3%) | 5 (18.5%) | 32 (17.1%) | 0.857 |
| Liver cirrhosis, n (%) | 115 (51.8%) | 17 (60.7%) | 98 (50.5%) | 0.313 |
| Tumor size (>2cm), n (%) | 175 (78.8%) | 21 (75.0%) | 154 (79.4%) | 0.596 |
| Tumor number (single : multiple) | 199 : 23 | 26 : 2 | 173 : 21 | 0.550 |
| Child-Pugh grade (A : B) | 201 : 21 | 26 : 2 | 175 : 19 | 0.654 |
| Micro/Macrovascular invasion, n (%) | 89 (40.1%) | 10 (35.7%) | 79 (40.7%) | 0.613 |
| Histological grade (well : moderate : poor) | 22 : 189 : 9 | 4 : 22 : 2 | 18 : 167 : 7 | 0.467 |
| Recurrence, n (%) | 137 (61.7%) | 20 (71.4%) | 117 (60.3%) | 0.258 |
| Death, n (%) | 63 (28.4%) | 14 (50.0%) | 49 (25.3%) | 0.007 |

AFP = α-fetoprotein
